# Measured outcomes of chronic care programs for older adults: a systematic review

**DOI:** 10.1186/s12877-015-0136-7

**Published:** 2015-10-26

**Authors:** Heather Drouin, Jennifer Walker, Heather McNeil, Jacobi Elliott, Paul Stolee

**Affiliations:** School of Public Health and Health Systems, University of Waterloo, 200 University Avenue West, Waterloo, ON N2L 3G1 Canada; School of Human and Social Development, Nipissing University, Muskoka Campus, 125 Wellington St, Bracebridge, ON P1L 1E2 Canada

**Keywords:** Chronic care model, Expanded chronic care model, Chronic illness, Seniors

## Abstract

**Background:**

Wagner’s Chronic Care Model (CCM), as well as the expanded version (ECCM) developed by Barr and colleagues, have been widely adopted as frameworks for prevention and management of chronic disease. Given the high prevalence of chronic illness in older persons, these frameworks can play a valuable role in reorienting the health care system to better serve the needs of seniors. We aimed to identify and assess the measured goals of E/CCM interventions in older populations. In particular, our objective was to determine the extent to which published E/CCM initiatives were evaluated based on population, community, system and individual-level outcomes (including clinical, functional and quality of life measures).

**Methods:**

We conducted a systematic search of the Science Citation Index Web of Knowledge search tool to gather articles published between January 2003 and July 2014. We included published CCM interventions that cited at least one of the fundamental papers that introduced and described the CCM and ECCM. Studies retained for review reported evaluations of senior-focused E/CCM initiatives in community-based settings, with the topic of “older adults” OR senior* OR elder* OR geriatric OR aged. The resulting 619 published articles were independently reviewed for inclusion by two researchers. We excluded the following: systematic reviews, meta-analyses, descriptions of proposed programs, and studies whose populations did not focus on seniors.

**Results:**

We identified 14 articles that met inclusion criteria. Studies used a wide range of measures, with little consensus between studies. All of the included studies used the original CCM. While a range of system-level and individual patient outcomes have been used to evaluate CCM interventions, no studies employed measures of population or community health outcomes.

**Conclusions:**

Future efforts to test E/CCM interventions with seniors would be aided by more consistent outcome measures, greater attention to outcomes for the caregivers of older persons with chronic illness, and a greater focus on population and community impacts.

## Background

Health care systems are frequently challenged by issues of access, continuity, fragmentation and quality of care in addressing the needs of older persons with chronic illness [1]. Wagner and colleagues [2] developed the Chronic Care Model (CCM) as a framework for the development of more comprehensive and integrated chronic care. The CCM framework includes six components: Community Resources and Policies; Health system organization; Self-Management Support; Decision Support; Delivery System Design; and Clinical Information Systems [2]. In this model, the community resources and health system components are designed to support engaged patients and proactive health care teams, which interact to improve functional and clinical outcomes for patients. Barr and colleagues [3] proposed an Expanded Chronic Care Model (ECCM) to support greater emphasis on population and community health outcomes.

The original and expanded versions of the Chronic Care Model (E/CCM) have been widely adopted [4]. We were interested in understanding the outcomes and indicators used to evaluate E/CCM interventions that focus on older adults with chronic illness. The objective of this paper was to determine the extent to which published E/CCM initiatives were evaluated based on population, community, system and individual-level outcomes (including clinical, functional and quality of life measures).

## Methods

We reviewed published studies of explicitly identified E/CCM interventions that included elements of the CCM model: self-management support, decision support, delivery system design, clinical information systems, health care organization, and community resources.

### Literature search

**Fig. 1:**
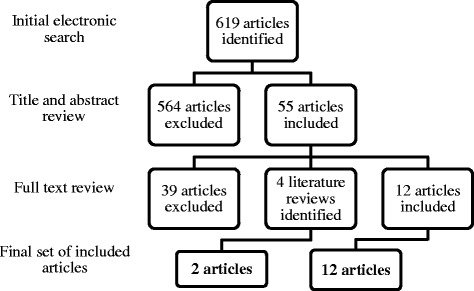
Literature Search Process

As described by Coleman and colleagues [5], the variation in nomenclature used by authors, and imprecisions in descriptions of interventions, can make it difficult to identify E/CCM interventions through usual database search strategies. In order to facilitate the identification of CCM-based interventions, Coleman and colleagues developed a strategy utilizing the Science Citation Index Web of Knowledge to limit their search to published interventions that cited at least one of the fundamental CCM papers [2, 6–9]. We utilized a similar strategy, but also identified interventions that cited the more recent paper by Barr and colleagues [3], which introduced the ECCM. We included articles published between January 2003 and July 2014, in English, with the topic of “older adults” OR senior* OR elder* OR geriatric OR aged. The search process is outlined in Fig. 1.

To be included, articles had to report an evaluation or observational study of an E/CCM intervention, and needed to examine the relationship between the intervention and clearly identified outcomes. We excluded the following: systematic reviews, meta-analyses, descriptions of proposed programs that lacked outcomes, and studies whose populations did not focus on seniors. The articles were independently reviewed for inclusion by two researchers; disagreements were resolved by consensus of the two reviewers or, if necessary, through group discussion among the authors.

As of July 2014, the search yielded 3630 articles that cited at least one of the six articles [2, 3, 6–9]; of these, 827 had a focus on older adults – 619 once duplicates were removed. After abstract and full text review, twelve articles were included in the final set. Of four literature reviews, one [10], was helpful in identifying two further articles for inclusion.

The resulting 14 included articles were reviewed to determine the level of reporting for each of the reported outcomes (population, community, health system, or individual) and the type of measure for each outcome (system impact, quality of care, or patient/caregiver outcome).

## Results

The included studies are summarized in Table 1. Most included studies focused on populations aged 65 (or 66 [11]) and older, except two which focused on persons aged 75+ [12, 13] and one with a wider age range (20–98) but an average age of 65.5 [14]. None of the papers in the final sample, even those published recently, used the ECCM as the basis of their intervention. Therefore, we did not anticipate that outcomes would be reported at the population level. However, consistent with the original CCM, we expected community level outcomes.

**Table 1:** Summary of included studies

[Study reference #]	Study design	Level of analysis		Type of measure		
Setting		Health System	Individual	System impact	Quality of Care	Individual outcome
		n = number of outcomes measured				
[18] Post-discharge from hospital	Longitudinal, Randomized trial	N/A	24 patient	Emergency Department visits	Quality of medical management	Cognitive function
			1 caregiver	Hospitalizations Nursing home admission	Patient involvement in decision making	Physical function
					Access to care	Quality of Life
					Satisfaction	ADL function
					Completion of advanced directives	IADL function
						Medications
						Blood pressure
						Depression and anxiety
						Falls
						Nutrition
						Pain
						Exercise
						Smoking
						Caregiver strain*
						Incontinence
						Knowledge of personal health risk factors
						Medication organization
						Disease management knowledge
[22] Community	Longitudinal, Randomized trial	N/A	5 patient	N/A	Symptom management	Quality of life/death
						Relationships
						Decision making/care planning/continuity/communication
						Depression and anxiety
[19] Integrated Services for Frail Elders (SIPA), Community Primary Hospital care	Randomized control trial	N/A	15 patient	Total healthcare costs	N/A	N/A
				Cost for community services		
				Cost for institutional services		
				Utilization of home care		
				Utilization of GP services		
				Number nursing home hours		
				Utilization of specialist care		
				Prescribed drugs		
				Number of days in acute care		
				Number of days in chronic care		
				Number of days in LTC facility		
				Number of hours of social services		
				Number of hours at ED		
				ED visits		
				Hospitalizations		
[16] Primary care practices	Cluster-randomized controlled trial	1	10 patient	Hospitalizations	Perceived quality of care	Caregiver depression*
			4 caregiver	Hospital days	Caregiver perceived quality of care*	Caregiver strain*
			2 provider	Skilled nursing facility admissions	Satisfaction with care^	
				Skilled nursing facility days	Satisfaction with knowledge^	
				ED visits	Team’s problem-solving performance#	
				GP visits		
				Specialist visits		
				Home healthcare		
				Healthcare costs		
				Productivity loss*		
[24] Primary care - capitated plan	Pilot implementation and evaluation	N/A	1 patient	N/A	Primary Care Assessment Survey (included communication, interpersonal treatment, knowledge of patient, integration of care, and trust in physician)	N/A
[23] Geriatric Ambulatory Practice	Longitudinal, Pre-post	N/A	7 patient	N/A	HBA1c test in last 9 months	HbA1c levels
					Foot examination done	LDL cholesterol level
					Lipid panel in last 9 months	Blood pressure
					LDL cholesterol test	
[14] Community	Mixed-methods (provider interviews and patient surveys)	1	3 patient	N/A	N/A	Physical quality of life
						Physical activity
						Current smoking
						DMP impact on healthier behavior#
[21] Hospital, community, Rehab centre, GP offices	Quality Improvement Project and evaluation	N/A	6 patient	N/A	GP opinion of collaboration^	Nutritional status
			1 provider		Patient satisfaction	Clinical tests
						Physical function
						Patient self-assessment of function
						Quality of life
[17] Primary care practices	Cluster randomized trial	1	10 patient	Acute hospital admissions	Patient reported client centred care	Quality of Life
			2 carer			
				Costs (direct and indirect costs)#	Coordination of care from patient perspective	Health-related Quality of Life
						Independence in ADL
						Psychological Wellbeing
						Social functioning
						Self-reported health
						Care needs
						Caregiver quality of life*
						Caregiver self-rated burden of care*
[20] Primary care - managed care plan	Longitudinal, Quasi experimental	3	2 patient	Health service resource use	N/A	N/A
				Cost of care		
				Number of hospitalizations#		
				Hospital bed days#		
				Number of ED visits#		
[13] Community	Randomized control trial	N/A	12 patient	Cost analysis	Perceived chronic illness care	Complexity of care needs
			2 caregiver	Service use	Self-management knowledge and behavior	Frailty
			1 provider			
					Impact of interventions	Health status
					Provider perceived chronic illness care^	Self-management ability
						Caregiver burden*
						Well-being
						Activities of daily living (ADL)
						Quality of life/*
[12] General Practice	Longitudinal, Quasi experimental (13 intervention practices, 11 control)	N/A	7 patient	Healthcare utilization	Care satisfaction	Health-related quality of life
				Nursing home admission		Disability in ADL and IADL
						Attitude towards aging
						Mortality
[15] Senior Health and Wellness Centre	Continuous quality improvement	3	7 patient	Service utilization#	Patient satisfaction	Clinical
					Diabetics with HbA1c less than 7	Function
					Chronic pain improvement	
					Employee satisfaction#	
					Pts with >4 meds receiving geriatric pharmacist review	
					Staff turnover rates#	
					Teamness#	
[11] Senior Health and Wellness Centre	Longitudinal panel	N/A	2 patient	N/A	N/A	Physical function
						Health-related quality of life

* = caregiver level measure

^ = provider level measure

# = system level measure

### Health system impacts

Health system impacts were considered in 12 of the 14 articles. Organization impacts were collected with three measures: employee satisfaction, staff turnover rates [15], and “teamness” [16]. Health service use, with seven distinct measures, was measured fairly uniformly through costs (direct/indirect) [17], emergency department visits [16, 18–20], hospitalizations and re-hospitalizations [16–20], hospital bed days [16], nursing home admission [12, 16, 18, 19], prescribed medications [19], and utilization of services (community, home care, specialist, etc.) [13, 15, 19, 20].

Quality of care measures were the most diverse (eighteen distinct measures) and were collected through three methods: provider perspectives of quality, patient perspectives of quality, and patient-related care processes. Professional caregiver measures of quality of care were assessed through perceptions of collaboration [21], quality of medical management [18] and impact of programs on health behaviour [14], as well as by using tools such as the Primary Care Assessment Survey [16], and the Assessment of Chronic Illness Care tool [13]. Quality of care from the patient’s perspective was more common, including measures of satisfaction [12, 15, 16, 18, 21], access to care [18], coordination of care [17], patient involvement in decision-making [18, 22], client-centred care [17], and provider performance [13]. Observed patient-related care processes included symptom/pain management [15, 18, 22], completion of advanced directives [18], appropriateness of tests performed (e.g., HbA1c, lipid panel, LDL cholesterol, pharmacist review, foot exam) [15, 23], appropriateness of biomedical test results [15, 22], and self-management knowledge/behaviour [13, 16, 18, 24].

### Individual impacts

Individual patient or caregiver outcomes were defined in 10 of the 14 articles. Patient outcomes were assessed using a wide range of measures. Biomedical measures were used in four studies [15, 18, 21, 23], and included blood pressure, HbA1c levels, LDL cholesterol level and nutritional status.

Functional status was assessed in seven studies [11–13, 15, 17, 18, 21] with measures of physical function, activities of daily living (ADL), instrumental ADL (IADL), cognitive function, and incontinence. Physical and functional (ADL/IADL) outcomes were collected using seven different methods, including the Groningen Activity Restriction Scale (by mail), Katz ADL index, Avlund Scale (self-assessment of physical function), Shuttle-walk test, chair stand test, 2.45 meter up and go, and a telephone administered physical function survey. Only one study included a personal indicator of frailty, in the form of the Groningen Frailty Index (GFI) self-report version.

Psychological wellbeing and mental health of patients was measured in four studies [13, 17, 18, 22] using measures of anxiety and depression, relationships, social functioning, and the Groningen Well-being Indicator (GWI). Seven studies [11–13, 17, 18, 21, 22] examined health-related quality of life through health status, quality of death, and level of pain. At least six instruments were used to measure quality of life: EQ-5D, SF-12, RAND-36, SF-36, 24 item HRQL from SF-36, and QUAL-E.

Five studies [12, 13, 16, 18, 22] measured patients’ knowledge, attitudes and abilities through various indicators, including attitudes towards aging, level of communication, decision-making capacity, knowledge of disease management and risk factors, and self-management ability. Two studies [14, 18] focused on health behaviours such as nutrition, smoking, exercise, and organization of medication. Patient care needs were measured in three studies [13, 18, 24] examining complexity of care needs, and medications. Adverse outcomes, specifically falls and mortality, were measured in two studies [12, 18].

Only four studies looked at the impact of CCM implementation on informal caregiver outcomes [13, 16–18]. These studies used a combination of measures of quality of life, burden of care, mental wellbeing and caregiver strain.

### Population/community impacts

None of the 14 papers reported outcomes measured at the population or community level. The level of analysis remained almost exclusively at the individual level (all articles included at least one measure collected at the individual level), while a minority (five articles) [14–17, 20], examined measures at the health system level.

## Discussion

Many published studies of E/CCM interventions lack detailed descriptions of the interventions evaluated and the study context, making it difficult to determine how closely the interventions correspond with the E/CCM frameworks. To ensure we included only studies specifically aimed at implementing elements of the E/CCM, we restricted our search to papers that cited one of the six foundational papers. The advantage of this is that we could appropriately examine the extent to which the outcomes reflected the E/CCM framework. However, this method may have excluded E/CCM interventions that did not include references to the original papers.

This paper provides a review of how CCM interventions in older populations are being evaluated for success and impact. As the CCM emphasizes the involvement of all levels of care to improve outcomes, it follows that outcomes would be measured at each of these levels. Several papers included measures of system impact, and all included measures of individual patient outcomes. Overall, there was a noticeable heterogeneity of outcomes measured in the studies, as well as in the associated methods and measurement instruments used. This lack of consistency in outcome measures is a common issue in evaluation of geriatric interventions and limits our ability to compare results across studies or to discern whether negative study results are due to an ineffective intervention or an inadequate measure [25]. Standardized health assessment and reporting systems could help to alleviate these concerns [25, 26].

We found a focus on patient outcomes, with very little focus on the supportive role of provider and informal caregivers, despite the importance of interactions and relationships between patients and their community partners [3]. There is an opportunity for future E/CCM based interventions to provide greater attention to quality of life and other outcomes for the caregivers of older persons with chronic conditions.

In the evaluations of CCM programs included in this review, no population or community outcomes were measured directly. The lack of studies using the newer ECCM was an interesting finding. The ECCM supports the design of services based on the needs and health characteristics of a population in order to improve an equitable distribution of health [27]. This is especially important for groups of patients with higher burdens of morbidity, such as older persons-with chronic illness. Our review suggests that while the ECCM may represent a significant conceptual advance, it has not yet guided empirical research that has resulted in peer-reviewed studies. This finding also highlights the need for greater integration of clinical programs with public and population health strategies. Others have also reflected on a lack of attention to the population and community-oriented elements of the E/CCM [1]. The impacts of some of the elements that may greatly affect disease, health, and quality of life (including patient support, system design, clinical decision support and clinical information systems) can be difficult to evaluate or measure, but may be of considerable importance to the overall success of E/CCM programs [1]. A recent scoping review on public health and primary care collaboration identified many potential barriers to collaboration, but also significant benefits for improved chronic care and disease prevention [28].

## Conclusions

The current literature on E/CCM interventions with older adults indicates that evaluation of these programs is often limited to health system performance indicators and clinical or functional outcomes for patients, all at the level of the individual. Outcomes are rarely measured at the health system level, and not at all at the population or community level. This review has identified a need for development of chronic care programs and related research that focus on population health or community impacts. An additional gap was found in the measurement of outcomes for caregivers, which is particularly relevant for programs that care for older persons with chronic illness, who frequently require support of family members or friends.

The CCM was developed to guide comprehensive system change [8, 29]; the ECCM [3] suggested an even broader scope. The World Health Organization’s adaptation of the CCM placed increased emphasis on its community and policy aspects [30]. Using the robust methods of a systematic review [31], we wished to explore whether the comprehensive aims of these models have been realized in their application and evaluation.

This and other recent reviews [32] have found that few studies of chronic care programs have addressed the community or policy components of these models. We believe this paper points to the need for more comprehensive chronic care prevention and management efforts. Similarly, there is a need for future efforts to support greater collaboration and integration across community and health system sectors, recognizing that this will come with significant challenges [33].

With more comprehensive approaches to intervention comes a need for more comprehensive approaches to evaluation and outcome measurement, which is another important implication of this paper. Advancement of chronic care research would benefit from more consistent frameworks and methods for outcome assessment. Work in other contexts may provide useful models to guide these efforts. Examples include the TOPICS-MDS initiative in the Netherlands to support consistent collection and sharing of data for research on health care of older persons [34], work of the OMERACT group related to rheumatology clinical trials [35], and efforts to identify consistent health outcome measures for older persons with multiple chronic conditions [36]. The monitoring and evaluation of coordinated, cross-system efforts would also benefit from consistent clinical information systems [37]. A consistent set of measures that could address outcomes at the health system, community and population levels would be of great value for future research.

Given the growing global burden of chronic disease, especially among a growing population of older persons [29, 38], we hope that our review will provide added impetus for more comprehensive prevention and management efforts, and more consistent approaches to their evaluation.

## Abbreviations

CCM: Chronic care model
ECCM: Expanded chronic care model
ADL: Activities of daily living
IADL: Instrumental activities of daily living
